# The impact of physical education teacher support on sport participation among college students: the chain mediating effects of physical education learning motivation and self-efficacy

**DOI:** 10.3389/fpsyg.2025.1592753

**Published:** 2025-06-02

**Authors:** Wenquan Su, Qiang Liu

**Affiliations:** School of Physical Education, Shandong University, Jinan, China

**Keywords:** college students, teacher support, learning motivation, self-efficacy, sports participation, chain mediation, physical education

## Abstract

**Objective:**

This study aimed to examine the relationship between physical education (PE) teacher support and sport participation among college students, with a particular focus on the mediating roles of PE learning motivation and self-efficacy.

**Methods:**

A cross-sectional survey was conducted using random sampling among 604 college students aged 18–29 years (354 males, 250 females; mean age = 20.44 ± 2.541 years) in Shandong Province, China. The study employed the Physical Education Teacher Support Scale, Physical Education Learning Motivation Scale, General Self-Efficacy Scale (GSES), and Sport Participation Scale. Data analyses were conducted using SPSS 27.0, PROCESS 3.5, and AMOS 24.0. Analyses included Harman’s single-factor test, reliability and validity assessments (Cronbach’s *α*, KMO, Bartlett’s test of sphericity, AVE, CR, CFA), descriptive statistics, Pearson correlation analysis, and mediation analysis using PROCESS Model 6.

**Results:**

Physical education teacher support, PE learning motivation, self-efficacy, and sport participation were all significantly positively correlated. The impact of PE teacher support on sport participation was mediated through three pathways: solely through PE learning motivation (mediating effect value: 0.146), solely through self-efficacy (mediating effect value: 0.166), and through the combined effect of PE learning motivation and self-efficacy (mediating effect value: 0.129).

**Conclusion:**

Physical education teacher support not only directly promotes college students’ sport participation but also indirectly facilitates participation by enhancing their PE learning motivation and self-efficacy.

## 1 Introduction

In recent years, insufficient physical activity has become a major issue affecting the physical and mental health of young people (World Health Organization, 2022). Although numerous studies have demonstrated that regular participation in physical activity brings a wide range of health benefits (Frederick et al., 1996), a considerable number of college students fail to engage in adequate exercise and thus miss out on these benefits. According to the Eighth National Survey on Student Physical Fitness and Health in China, although improvements were observed in some age groups, declining physical fitness and insufficient sports participation among college students remain particularly prominent and warrant urgent attention (Department of Physical Health and Arts Education Ministry of Education, 2021). Furthermore, research on the factors influencing sport participation among college students remains relatively limited, especially within the Chinese cultural context. Existing studies have not yet explored effective strategies for promoting sport participation among Chinese college students in depth. Therefore, investigating the impact of physical education (PE) teacher support on sport participation in this population holds important academic and practical significance.

In the Chinese higher education context, PE courses are typically elective, and students’ motivation and confidence to participate in sports are greatly influenced by teacher support. As an external motivating factor, teacher support can significantly increase students’ engagement and enthusiasm for sport participation (Yu et al., 2021). Prior research has shown that teachers play not only a central role in the transmission of academic knowledge but also a critical role in stimulating student motivation and shaping attitudes and behaviors. Students’ perceptions of teacher support affect key psychological factors such as goal orientation and achievement motivation, and positive teacher-student interactions can significantly enhance students’ cognitive, emotional, and behavioral outcomes (Eberline et al., 2018; Ghorbani et al., 2020).

The college years are a critical period for physical and mental development and for forming lifelong habits. Participation in sports not only improves physical fitness but also enhances mental health, stress management, and social skills. Therefore, encouraging college students to actively participate in physical activity is crucial for their holistic development and long-term well-being. Current research on college sport participation mainly falls into two categories: studies using longitudinal data to explore causal relationships (Kruse et al., 2024) and studies examining the influence of internal and external factors such as teacher and peer support (Van Boekel et al., 2016). Although these studies have contributed to understanding how to promote sport participation, most have focused on individual-level factors and have rarely examined the specific role of PE teacher support. Consequently, there is an urgent need for further research on how PE teacher support influences college students’ sport participation, particularly through its effects on students’ learning motivation and self-efficacy.

Based on these considerations, this study integrates PE teacher support and college student sport participation into a unified model to explore their underlying relationships, mechanisms, and pathways. By revealing the chain mediating effects of teacher support, learning motivation, and self-efficacy, this study aims to provide valuable insights for promoting active engagement in physical activity among college students, enhancing participation levels, and fostering their physical and mental health.

## 2 Theoretical framework and research hypotheses

### 2.1 Physical education teacher support and sports participation

Physical education teacher support is considered an important factor influencing students’ sports participation (Guo et al., 2023). Research has shown that teachers can effectively stimulate students’ interest in sports and motivation to participate by providing emotional support, skill guidance, and creating a positive learning environment (Alcalá and Garijo, 2017). Chatzisarantis and Hagger conducted a large-scale intervention study that confirmed the positive effect of teacher support on students’ participation in extracurricular sports activities. Compared to students in neutral teacher support conditions, those receiving teacher support demonstrated stronger exercise intentions and more frequent participation in extracurricular sports (Chatzisarantis and Hagger, 2009). Especially at the college level, where most physical education courses are electives, students’ motivation to participate relies more on teacher support and encouragement rather than external coercion. By offering personalized guidance and positive interactions, physical education teachers can significantly enhance students’ interest in sports, thereby promoting their long-term participation (Zalech, 2021). In addition, teacher support helps students believe they can overcome challenges during participation, thereby strengthening their determination to stay engaged. Positive teacher support provides students with confidence, enabling them to face challenges and maintain an active participation attitude. Furthermore, in addition to sports settings, teachers’ instructional behaviors and attention to students are also related to students’ learning motivation and classroom engagement. However, the mutual influence between teacher support and student sports participation still lacks further exploration. This study proposes the first hypothesis:

*H1*: Physical education teacher support significantly and positively predicts college students’ sports participation.

### 2.2 The mediating role of physical education learning motivation

Physical education (PE) learning motivation refers to the internal drive that compels individuals to engage in sports activities based on intrinsic interest, emotional experiences, and personal goals. According to Self-Determination Theory (SDT), individuals’ behavioral motivation is shaped by both intrinsic and extrinsic factors, with teacher support playing a crucial role in fostering students’ intrinsic motivation and enhancing their sense of autonomy (Deci and Ryan, 1985, 2000). In the context of PE, teacher support—including emotional encouragement, skill instruction, and the creation of a positive learning environment—can significantly increase students’ intrinsic motivation, thereby stimulating their interest and drive to participate in sports activities (Ulstad et al., 2019). As a key mediating variable, PE learning motivation moderates the relationship between PE teacher support and students’ actual participation in sports (Leo et al., 2022). Research has shown that teacher support not only boosts students’ interest in physical activities but also strengthens their long-term commitment, with this heightened motivation serving as a critical factor for sustained participation (Otundo and Garn, 2019; Pluta et al., 2020). Therefore, PE learning motivation is hypothesized to mediate the relationship between PE teacher support and sports participation by transforming teacher support into meaningful behavioral engagement. Based on this, the following hypothesis is proposed:

*H2*: Physical education learning motivation mediates the relationship between physical education instructor support and sport participation among college students.

### 2.3 The mediating role of self-efficacy

Self-efficacy refers to an individual’s confidence in their ability to successfully complete a task, particularly when facing challenges. Social Cognitive Theory (SCT) emphasizes that self-efficacy plays a pivotal role in shaping behavior, as it directly affects individuals’ motivation, actions, and decision-making (Bandura, 1997). According to this theory, students’ self-efficacy is significantly influenced by their intrinsic motivation. Specifically, PE learning motivation can boost students’ confidence, leading to greater engagement and persistence in sports activities (Voskuil and Robbins, 2015). In the context of PE, teachers’ emotional support, skill instruction, and positive feedback can stimulate students’ learning motivation, which in turn enhances their self-efficacy. Studies have shown that when students perceive strong teacher support, their self-efficacy improves, making them more likely to actively participate in physical activities (Sánchez-García et al., 2024). Self-efficacy occupies a central position in predictive models of sports participation and has been found to be positively associated with PE teacher support (Ke and Huang, 2023). Based on this evidence, the following hypothesis is proposed:

*H3*: Self-efficacy mediates the relationship between physical education faculty support and sport participation among college students.

### 2.4 The chain mediation of physical education learning motivation and self-efficacy

Research has shown that sports participation may be influenced by both PE learning motivation and self-efficacy, and all three are closely related to PE teacher support (Yu and Song, 2022). Moreover, self-efficacy is shaped by learning motivation. Social theory suggests that self-efficacy is critical to individuals’ learning behaviors and academic achievement because it profoundly impacts motivation and emotions (Bandura, 1989). Students with high self-efficacy are typically more confident in pursuing academic goals and have stronger expectations for improving their abilities and performance, which leads to greater investment of time and effort in learning (Anam and Stracke, 2016). Expectancy-value theory also highlights the direct relationship between learning motivation and self-efficacy (Wigfield and Eccles, 2000). Furthermore, empirical studies have found that self-efficacy is not only positively correlated with learning motivation but also serves as a protective factor for sustaining it (Cetin-Dindar, 2015; Wu et al., 2020). Additionally, intrinsic exercise motivation has been shown to significantly predict individuals’ exercise self-efficacy (Neace et al., 2022).Based on the above research evidence, this study hypothesizes that there may be an indirect effect pathway from physical education teacher support → physical education learning motivation → self-efficacy → sports participation. By enhancing students’ learning motivation and self-efficacy through teacher support, sports participation can be effectively promoted. Therefore, this study proposes the fourth hypothesis:

*H4*: Physical education motivation and self-efficacy act as chain mediators between physical education instructor support and sport participation among college students.

**Figure 1:**
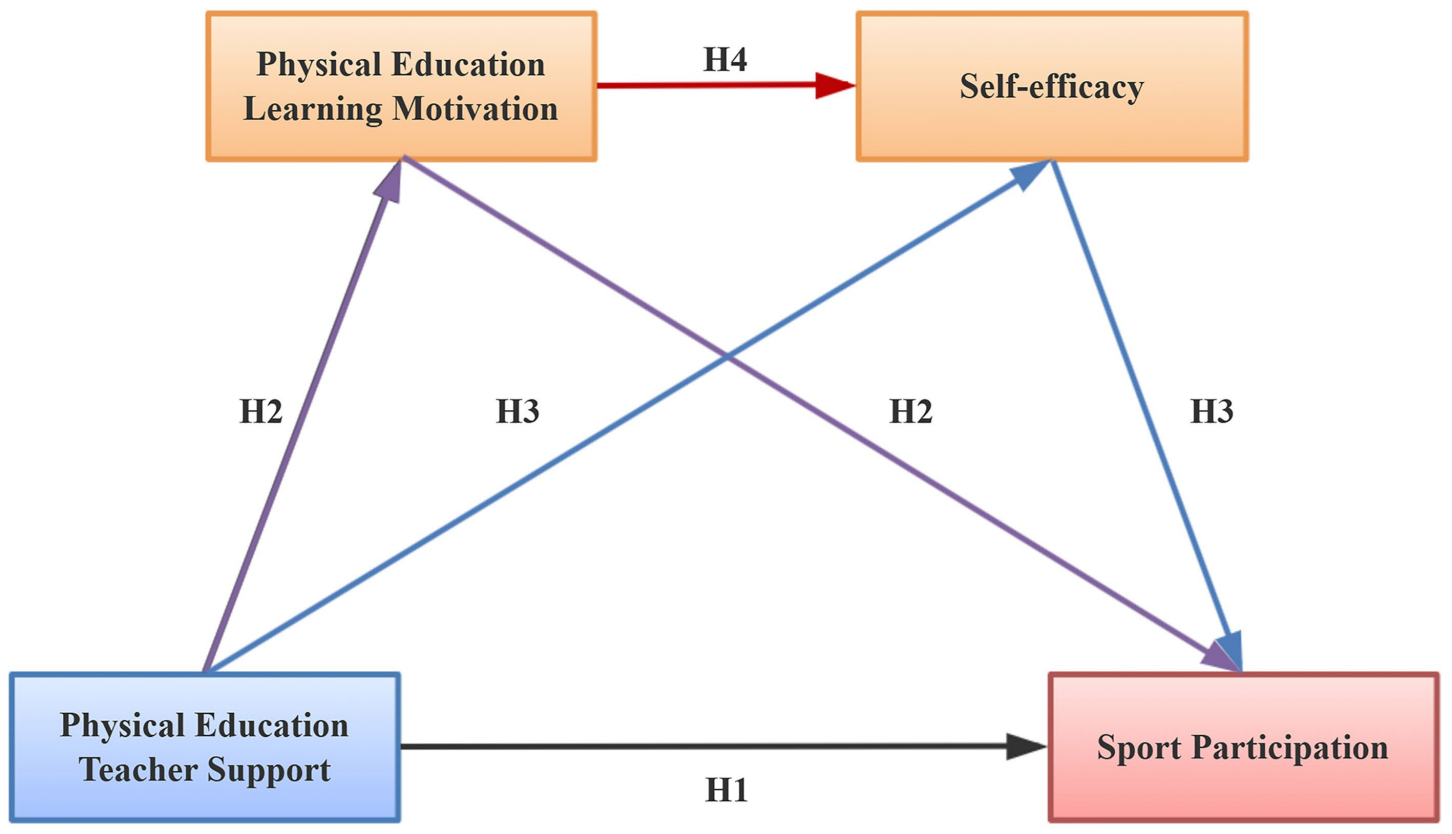
Hypothetical model.

In summary, this study focuses on Chinese college students and aims to examine the relationship between PE teacher support and sports participation within the context of public physical education practice courses. It also seeks to investigate whether PE learning motivation and self-efficacy mediate this relationship. As illustrated in Figure 1, this study proposes a hypothesized model to uncover the underlying mechanisms through which PE teacher support influences college students’ sports participation, thereby providing theoretical guidance for the development of effective intervention strategies and sports participation promotion programs.

## 3 Materials and methods

### 3.1 Participants

This study employed a random sampling method. Between January 6 and January 24, 2025, participants were randomly selected from five universities in Shandong Province, China. Building on convenience sampling, a random number table was used to randomly select students from 25 preselected classes to participate in the survey. The participants included full-time undergraduate students from first to fifth year, as well as master’s and doctoral students, primarily between the ages of 18 and 29. All participants had enrolled in public physical education practice courses designed for non-PE majors, such as soccer, basketball, or tennis, and voluntarily agreed to take part in the study. Data collection was conducted via the online platform Wenjuanxing using electronic questionnaires. A total of 702 questionnaires were distributed. To ensure data quality, exclusion criteria for invalid questionnaires were established, including incomplete demographic information, logically inconsistent responses, over 80% identical answers, more than 10 missing items, responses inconsistent with the question intent, and multiple selections for single-choice questions. After screening, 604 valid questionnaires were obtained, resulting in a valid response rate of 86.04%.

Among the valid questionnaires, 354 participants were male (58.61%) and 250 were female (41.39%). The distribution by academic level was as follows: 208 freshmen (34.44%), 147 sophomores (24.34%), 78 juniors (12.91%), 76 seniors (12.58%), 5 fifth-year students (0.83%), 50 master’s students (8.28%), and 40 doctoral students (6.62%). Regarding place of origin, 292 participants (48.34%) were from urban areas and 312 (51.66%) were from rural areas. In terms of family background, 206 participants (34.11%) were only children, while 398 (65.89%) had siblings. The participants ranged in age from 18 to 29 years, with 62.42% between 18 and 20 years old, 24.17% between 21 and 23, 8.94% between 24 and 26, and 4.47% between 27 and 29. All respondents were adults (≥18 years old), with a mean age of 20.44 ± 2.541 years (M ± SD). Demographic characteristics of the participants are presented in Table 1.

**Table 1:** Demographic characteristics of the participants.

Demographic variables	Category	*N*	Percentage
Gender	Male	354	58.61%
	Female	250	41.39%
Only-child status	Yes	206	34.11%
	No	398	65.89%
Age	18–20	377	62.42%
	21–23	146	24.17%
	24–26	54	8.94%
	27–29	27	4.47%
Birthplace	Urban	292	48.34%
	Rural	312	51.66%
Grade	Freshman	208	34.44%
	Sophomore	147	24.34%
	Junior	78	12.91%
	Senior	76	12.58%
	Fifth-year students	5	0.83%
	Master’s students	50	8.28%
	Doctoral students	40	6.62%

### 3.2 Measures

#### 3.2.1 Physical education teacher support

Physical education teacher support was measured using the Teacher Support Scale developed by Chi (2017), which was later revised into the Physical Education Teacher Support Scale by Zhang (2024). This scale consists of 12 items covering three dimensions: autonomy support, emotional support, and competence support. A 5-point Likert scale was used (1 = completely disagree, 5 = completely agree), with higher total scores indicating greater perceived support from physical education teachers. Previous research has demonstrated that the scale has good applicability and reliability among Chinese college students (Zuo, 2022). In the present study, the scale demonstrated a Cronbach’s *α* coefficient of 0.972.

#### 3.2.2 Physical education learning motivation

The Physical Education Learning Motivation Scale was originally developed by Guay and Vallerand (1996) and later adapted for use in China by Su (2007). The revised version has been validated among Chinese college students and has demonstrated good cultural adaptability (Wei and Hong, 2009). This scale includes 14 items covering four dimensions: intrinsic motivation, external regulation, identified regulation, and amotivation. A 5-point Likert scale was used (1 = completely inconsistent, 5 = completely consistent), with higher total scores indicating stronger physical education learning motivation. In the present study, the scale showed a Cronbach’s *α* coefficient of 0.913.

#### 3.2.3 Self-efficacy

The General Self-Efficacy Scale (GSES) was the version revised by Wang et al. (2001). This version has been widely used and validated among Chinese college student samples, demonstrating good reliability and validity (Chen et al., 2024). The scale consists of 10 items and uses a 4-point Likert scale (1 = completely inconsistent, 4 = completely consistent), with a total score ranging from 0 to 40. Higher scores indicate stronger self-efficacy. In the present study, the Cronbach’s *α* coefficient for the scale was 0.968.

#### 3.2.4 Sports participation

The Physical Activity Rating Scale (PARS-3) was developed by Liang (1994), and Lu later revised it to create the Sports Participation Scale (Lu, 2021). The revised version has been widely used in studies targeting Chinese college students and has demonstrated good cultural applicability (Li et al., 2023). The scale consists of 11 items, covering three dimensions: behavioral participation, emotional participation, and cognitive participation in sports. A 5-point Likert scale is used to calculate the total score, with higher scores indicating higher levels of sports participation. In the present study, the Cronbach’s α coefficient for this scale was 0.923.

### 3.3 Reliability and validity testing of the questionnaire

The reliability of the study variables was analyzed using SPSS 27.0. The results showed that all variables had Cronbach’s α coefficients above 0.90, indicating high internal consistency and reliability of the scales. The KMO value was 0.968, and the Bartlett’s test of sphericity was significant (*p* < 0.05), suggesting that the data were suitable for further factor analysis. The Average Variance Extracted (AVE) ranged from 0.655 to 0.909, and Composite Reliability (CR) values all exceeded 0.90, collectively indicating good convergent validity. Detailed results of the reliability and validity tests are presented in Table 2.

**Table 2:** Results of scale reliability test.

Variable	Number of Items	Cronbach’s α	AVE	CR
Physical education teacher support	12	0.972	0.909	0.968
Physical education learning motivation	14	0.913	0.758	0.925
Self-efficacy	10	0.968	0.754	0.968
Sports participation	11	0.923	0.655	0.842

The evaluation of discriminant validity was based on the results of Confirmatory Factor Analysis (CFA) conducted using AMOS 24.0. CFA is a commonly used statistical method to verify the factor structure of observed variables and is an important tool for testing the validity of measurement models (Kline, 2005; Byrne, 2013). During the evaluation process, selecting appropriate model fit indices is essential. The fit indices used in this study included: χ^2^/df, Comparative Fit Index (CFI), Tucker-Lewis Index (TLI), Goodness of Fit Index (GFI), Normed Fit Index (NFI), Incremental Fit Index (IFI), Standardized Root-Mean-Square Residual (SRMR), and Root-Mean-Square Error of Approximation (RMSEA). The CFA results are shown in Table 3.

**Table 3:** Validation of factor analysis goodness-of-fit results.

Fit Index	χ^2^/df	CFI	TLI	GFI	NFI	IFI	SRMR	RMSEA
Reference value	<5.000	>0.900	>0.900	>0.900	>0.900	>0.900	<0.080	<0.080
Test value	4.519	0.955	0.948	0.868	0.943	0.955	0.053	0.076

According to the evaluation criteria proposed by Byrne (2013), the model showed good fit: χ^2^/df = 4.519, CFI = 0.955, TLI = 0.948, GFI = 0.868, NFI = 0.943, IFI = 0.955, SRMR = 0.053, and RMSEA = 0.076. A χ^2^/df value below 5 is considered acceptable. Although GFI was slightly below 0.900, the other indices met the standards for good fit. CFI, TLI, NFI, and IFI all exceeded 0.900, and RMSEA and SRMR were below 0.080, indicating that the measurement model had good discriminant validity overall (Steiger, 1990; Hu and Bentler, 1999; Schreiber et al., 2006).

### 3.4 Check for common method bias

Since data in this study were collected through an online questionnaire, it was necessary to test for potential common method bias (CMB). First, procedural remedies such as the inclusion of reverse-coded items and anonymous responses were employed during the survey to reduce the risk of common method bias. Second, Harman’s single-factor test was conducted to assess common method bias. The results showed that five factors had eigenvalues greater than one, and the variance explained by the first common factor was 38.85%, which is below the critical threshold of 40%, indicating that significant common method bias was not present in this study (Podsakoff et al., 2003).

### 3.5 Data analysis

Data analysis in this study was conducted using SPSS 27.0, AMOS 24.0, and Hayes’ PROCESS macro 3.5. First, the Kaiser-Meyer-Olkin (KMO) test and Bartlett’s test of sphericity were performed to assess the suitability of the data for factor analysis. The KMO value was 0.968, and Bartlett’s test was significant (*p* < 0.05), indicating that the data were appropriate for subsequent factor analysis. Next, Harman’s single-factor test was used to examine common method bias, and the results showed no serious bias. Descriptive analyses were then conducted to summarize participants’ demographic characteristics. Cronbach’s *α* coefficients were calculated using SPSS, all of which exceeded the 0.90 threshold, indicating good reliability of the scales. Average Variance Extracted (AVE) and Composite Reliability (CR) were calculated using AMOS, both meeting the requirements for validity testing. To verify structural validity, a Confirmatory Factor Analysis (CFA) was performed, and the model showed good fit, with indices such as χ^2^/df, CFI, TLI, NFI, IFI, RMSEA, and SRMR all meeting conventional standards. Pearson correlation analysis was conducted to assess associations between variables. Finally, mediation effects were tested using Hayes’ PROCESS macro, employing bootstrapping with 5,000 samples to calculate 95% confidence intervals (CI) (Shrout and Bolger, 2002). A mediation effect was considered significant if the 95% CI did not include zero.

## 4 Results

### 4.1 Descriptive statistics and correlations

Pearson correlation analysis was used to examine the relationships among age, gender, physical education teacher support, physical education learning motivation, self-efficacy, and sports participation. As shown in Table 4, all key study variables were positively correlated.

**Table 4:** Descriptive statistics and intercorrelations among variables.

Variable	*M*	*SD*	1	2	3	4	5	6
1. Gender	1.41	0.49	–					
2. Grade	2.72	1.88	0.316**	–				
3. PETS	46.62	11.43	−0.113**	−0.344**	–			
4. PELM	51.68	11.42	−0.056	−0.141**	0.456**	–		
5. SE	32.91	7.18	0.040	0.030	0.343**	0.458**	–	
6. SP	61.75	31.94	0.144**	0.342**	0.172**	0.254**	0.387**	–

PETS, physical education teacher support; PELM, physical education learning motivation; SE, self-efficacy; SP, sports participation; *p < 0.05; **p < 0.01; ***p < 0.001.

### 4.2 The mediating effects analysis

Previous studies have shown that gender and age can influence college students’ mental health (Liu et al., 2023). Therefore, gender and grade level were included as control variables to test the mediating roles of physical education learning motivation and self-efficacy in the relationship between physical education teacher support and sports participation among college students. Model 6 of the SPSS PROCESS macro 3.5 by Hayes was used, with 5,000 bootstrap samples and 95% confidence intervals (CI) computed. The regression results are presented in Table 5: physical education teacher support significantly positively predicted sports participation (*β* = 0.918, *p* < 0.001), physical education learning motivation (*β* = 0.462, *p* < 0.001), and self-efficacy (*β* = 0.142, *p* < 0.001). Physical education learning motivation significantly positively predicted self-efficacy (*β* = 0.238, *p* < 0.001). When physical education teacher support, physical education learning motivation, and self-efficacy were entered simultaneously to predict sports participation, both physical education learning motivation (*β* = 0.317, *p* < 0.01) and self-efficacy (*β* = 1.170, *p* < 0.001) showed significant positive effects on sports participation. At this point, the positive predictive effect of physical education teacher support on sports participation remained significant (*β* = 0.478, *p* < 0.001), indicating that the chain mediating roles of physical education learning motivation and self-efficacy were supported.

**Table 5:** Results of intermediary model regression analysis.

Outcome variable	Predictor variable	*R*	*R* ^2^	*F*	β	*t*
SP	PETS	0.463	0.214	54.412***	0.918	8.526***
	Gender				2.651	1.072
	Grade				7.538	10.959***
PELM	PETS	0.456	0.208	52.608***	0.462	11.947***
	Gender				−0.245	−0.276
	Grade				0.132	0.533
SE	PETS	0.507	0.257	51.737***	0.142	5.403***
	PELM				0.238	9.561***
	Gender				0.585	1.082
	Grade				0.567	3.766***
SP	PETS	0.549	0.301	51.514***	0.478	4.124***
	PELM				0.317	2.744**
	SE				1.170	6.629***
	Gender				2.112	0.904
	Grade				6.797	10.338***

PETS, physical education teacher support; PELM, physical education learning motivation; SE, self-efficacy; SP, sports participation; *p < 0.05; **p < 0.01; ***p < 0.001.

**Figure 2:**
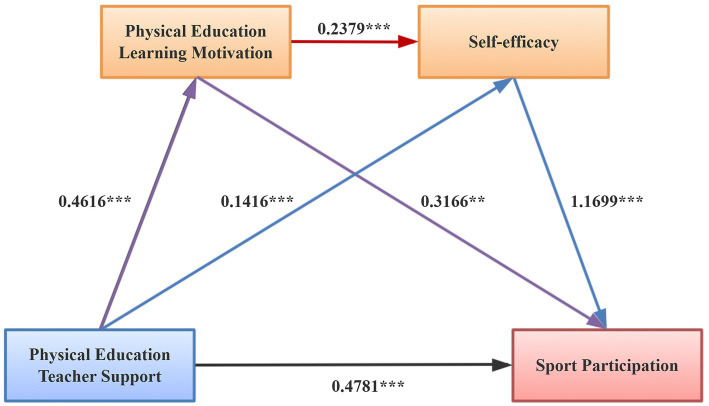
Regression and chain mediation models (*p < 0.05, **p < 0.01, and ***p < 0.001).

Further mediation analysis results are shown in Table 6 and Figure 2. The direct effect of physical education teacher support on sports participation was 0.478, with a confidence interval excluding zero, indicating a significant direct effect and accounting for 52.06% of the total effect. The total indirect effect was 0.440, also with a confidence interval excluding zero, indicating that the mediating effects of physical education learning motivation and self-efficacy were significant, accounting for 47.94% of the total effect. Specifically, the indirect path through physical education learning motivation alone had an effect size of 0.146, 95% CI = [0.012, 0.300]; through self-efficacy alone, the effect size was 0.166, 95% CI = [0.074, 0.284]; and through the chain mediation of physical education learning motivation and self-efficacy, the effect size was 0.129, 95% CI = [0.072, 0.202]. These results indicate that all three indirect paths were statistically significant.

**Table 6:** Mediation effect test results.

Model effect	Effect size	Boot SE	95%CI		Effect proportion
			LLCI	ULCI	
Total effect	0.918	0.108	0.707	1.130	
Direct effect: PETS → SP	0.478	0.116	0.250	0.706	52.06%
Total indirect effect	0.440	0.078	0.296	0.601	47.94%
PETS → PELM → SP	0.146	0.073	0.012	0.300	15.92%
PETS → SE → SP	0.166	0.054	0.074	0.284	18.04%
PETS → PELM → SE → SP	0.129	0.033	0.072	0.202	13.99%

PETS, physical education teacher support; PELM, physical education learning motivation; SE, self-efficacy; SP, sports participation.

## 5 Discussion

### 5.1 The impact of physical education teacher support on sports participation among college students

The results showed a significant positive correlation between physical education teacher support and sports participation among college students. Even after accounting for the mediating variables, the positive predictive effect of teacher support on sports participation remained significant, indicating that as the level of perceived teacher support increased, the level of sports participation also significantly rose, thus supporting Hypothesis 1. This finding is consistent with prior research. For example, Koka and Hein found that teacher support significantly enhances students’ intrinsic motivation, which in turn effectively promotes their engagement in physical activity (Koka and Hein, 2006). Physical education teacher support influences students’ sports participation through multiple mechanisms. First, positive feedback and appropriate challenges from teachers can enhance students’ perceptions of their own abilities, aligning with Bandura’s self-efficacy theory, which emphasizes that individuals’ confidence in their abilities is a key driver of behavioral engagement (Bandura, 1977). When students receive positive reinforcement, their self-confidence increases, which further stimulates their willingness to participate. Additionally, when teachers design activities matched to students’ skill levels, students experience a sense of achievement within an appropriate level of challenge, helping to spark interest, reduce fear of failure, and enhance their sense of safety and willingness to persist in sports activities.

Moreover, teacher support can help students select sports that align with their ability levels, thereby creating a more positive learning and participation environment. This finding underscores that the role of physical education teachers goes beyond merely delivering instructional content—they should also focus on building supportive relationships that help students overcome self-doubt, strengthen their sense of engagement, and foster a sense of accomplishment in sports participation.

In terms of teacher training and curriculum design, it is essential to train teachers on how to provide targeted support tailored to individual student differences. Teacher training should focus on improving teachers’ sensitivity to student needs and their ability to apply supportive strategies, covering not only physical skill instruction but also psychological support and emotional management, thereby motivating students to participate more actively in sports. Curriculum design should offer diverse sports options that align with students’ skill levels and interests, ensuring that each student can experience satisfaction and accomplishment in activities suited to their abilities. Through this personalized teaching approach, students’ self-confidence and interest in sports can be enhanced, ultimately encouraging long-term engagement in physical activity and leading to deeper psychological and physical benefits.

### 5.2 The independent mediating role of physical education learning motivation

The results showed that physical education teacher support not only directly and positively predicted college students’ sports participation but also indirectly influenced participation through the independent mediating role of physical education learning motivation, supporting Hypothesis 2. This finding aligns with Miller’s research, which, based on self-determination theory, emphasizes that intrinsic motivation plays a central role in individuals’ engagement in various activities and highlights that intrinsic motivation is the most enduring form of motivation, fostering enjoyment and self-regulation (Miller et al., 1996). This theoretical framework provides an important perspective for understanding how students can transform teacher support into learning motivation, thereby enhancing sports participation.

Previous research has also confirmed a significant positive correlation between students’ physical education learning motivation and their sports participation. Studies have shown that college students with high levels of learning motivation are more likely to engage in sports activities (Liu and Li, 2023). Frikha’s study further demonstrated that in the context of physical education, students’ motivation, enjoyment, and BMI act as critical mediators between extracurricular sports activities and academic achievement, underscoring the central role of motivation and emotional experience in sports participation (Frikha, 2025). This suggests that learning motivation not only serves as a positive feedback response to teacher support but also further influences students’ sports participation behaviors. During the physical education process, teacher support provides a vital external driving force, which is reflected not only in task assignment, evaluation, and incentives but also in emotional support, feedback, and guidance, all of which exert a subtle yet positive influence on students’ sports behaviors. As Deci and Ryan noted, appropriate teacher support can effectively enhance students’ intrinsic motivation, thereby boosting their enthusiasm and persistence in exercise (Deci and Ryan, 2000). Bordbar’s research further highlighted that autonomy-supportive teaching can stimulate students’ positive academic emotions and self-system processes, thereby improving their learning motivation and proactive engagement (Bordbar, 2021). When students perceive teacher support, they are more likely to translate that experience into a deeper understanding of physical activity, engage with a more positive attitude, and gain intrinsic satisfaction.

The data analysis in this study further validated these theoretical perspectives, showing a significant mediating effect of physical education learning motivation between teacher support and sports participation. This finding reveals the complex and close interaction between teacher support and students’ learning motivation in the educational environment, as well as how they work together to promote students’ sports participation. This process highlights not only the close connection between physical education and students’ cognitive and emotional development but also the pivotal role of teacher support in shaping students’ attitudes and experiences toward sports activities. Based on this conclusion, physical education should not only emphasize the role of teacher support but also pay attention to subjective factors such as learning motivation as mediators of sports participation. To achieve this, systematic teacher training is needed to improve teachers’ psychological and emotional support skills, enabling them to more effectively identify and stimulate students’ motivation during the learning process. Additionally, curriculum design should incorporate students’ autonomy and diverse needs, offering challenging and choice-based sports programs, introducing group collaboration, competitions, and phased goal setting to enhance students’ sense of achievement and belonging. Integrating activities like self-reflection, peer evaluation, and self-directed training plans can further deepen students’ understanding and intrinsic identification with physical activity. By balancing teacher support with student autonomy, curriculum design can effectively stimulate learning motivation and help translate it into sustained sports participation.

### 5.3 The independent mediating role of self-efficacy

The results indicated that physical education teacher support not only directly and positively predicted college students’ sports participation but also indirectly influenced participation through the independent mediating role of self-efficacy, supporting Hypothesis 3. In physical education settings, teachers help enhance students’ self-efficacy, which refers to their confidence in completing physical tasks and overcoming challenges, through positive feedback, encouragement, and appropriate levels of challenge. Previous research has shown that when students believe they have the ability to succeed in physical activities, they are not only more willing to try but also more likely to persist, ultimately improving their level of participation (Ouyang et al., 2020). This study found that when students perceived effective teacher support, their self-efficacy significantly increased, and this confidence, in turn, strengthened their engagement and persistence when facing physical challenges, promoting more stable and sustained participation.

Bandura’s research emphasized that college students’ self-efficacy is closely linked to their level of sports participation and can significantly predict the persistence of their exercise behaviors (Bandura, 1977), which is highly consistent with the present study’s findings. Specifically, teacher support involves more than just skill instruction and technical correction; it also includes emotional encouragement and the recognition of student effort. When teachers provide specific and positive feedback in physical education classes and design activities with appropriate challenges and opportunities for success, students’ confidence increases, which leads to greater enthusiasm for participation. Further analysis showed that self-efficacy serves as a significant mediator between physical education teacher support and sports participation. By providing multiple layers of support, teachers help students build confidence and a sense of control, which facilitates higher levels and longer-lasting engagement in sports activities.

The findings of this study highlight the importance of how teachers can foster and develop students’ self-efficacy to further encourage sports participation within teaching and curriculum design. Courses should focus on offering diverse and challenging activities that help students experience achievement and, in doing so, strengthen their self-efficacy and motivation for exercise. Furthermore, these findings emphasize the important role of teachers in shaping students’ psychological expectations and behavioral patterns. Future teacher training programs should focus on improving teachers’ support skills, including how to use encouragement, positive feedback, and effective guidance to strengthen students’ self-efficacy. By incorporating demonstration teaching, role-playing, and reflective exercises, teachers can become more proficient at applying personalized support strategies in practice, thereby enhancing students’ enthusiasm and persistence in sports participation. Overall, physical education teachers should aim to create a supportive and motivating classroom atmosphere that uses positive feedback and appropriate challenges to promote active participation and the long-term development of healthy exercise habits.

### 5.4 The chain mediating role of physical education learning motivation and self-efficacy

The results showed that physical education learning motivation and self-efficacy played a chain mediating role between physical education teacher support and college students’ sports participation, supporting Hypothesis 4. This study revealed how teacher support enhances students’ physical education learning motivation, which in turn strengthens their self-efficacy and ultimately promotes their participation in sports. This finding offers a new perspective for effectively encouraging sports participation among college students. Previous studies have shown that students with higher learning motivation typically exhibit stronger self-efficacy (Dong et al., 2023). This aligns with Liu et al.’s findings that teachers’ autonomy-supportive behaviors can enhance students’ autonomous motivation and positive emotions, thereby improving their self-efficacy (Liu et al., 2021). Similarly, this study found that when teachers provided support, students’ learning motivation significantly improved, which then encouraged them to engage more actively in sports. These results suggest that teacher support is not limited to technical instruction but also includes emotional support and feedback, which together effectively enhance students’ learning motivation and self-efficacy.

The study also highlighted the interactive relationship between physical education learning motivation and self-efficacy. Students with higher learning motivation are generally able to develop stronger self-efficacy because they are more actively involved in sports activities, gaining a greater sense of achievement and confidence. This active participation not only strengthens their belief in their own abilities but also helps them overcome challenges, further enhancing their self-efficacy. The chain mediation model presented here illustrates the complexity of physical education: teacher support first boosts students’ learning motivation, which then increases their self-efficacy, ultimately leading to more active engagement in sports. This finding provides a theoretical foundation for physical education practices and underscores the importance of fostering both learning motivation and self-efficacy in the teaching process.

In summary, teacher support, students’ physical education learning motivation, and self-efficacy are crucial determinants of sports participation among college students. This suggests that future physical education programs should prioritize this chain mediation mechanism, encouraging teachers to adopt more personalized and supportive instructional strategies to help students overcome difficulties in sports activities and strengthen their willingness and persistence to participate. Doing so will not only improve students’ physical fitness but also promote their psychological well-being and social adaptability, thereby contributing to the achievement of holistic educational goals.

## 6 Research implications and limitations

### 6.1 Research implications

Based on the results and discussion, several key implications can be drawn regarding college students’ participation in physical activity.

In physical education, teachers are not only responsible for delivering instructional content and providing basic support but should also foster an exercise environment that enhances students’ sense of experience and achievement through ability-based feedback and positive guidance. Of particular importance, the role of teacher support should not be limited to external environmental factors but should also address students’ subjective factors, such as physical education learning motivation, to effectively promote participation.

Additionally, heuristic teaching strategies can be incorporated into instruction to help students develop the ability to analyze, adjust, and reflect on their sports skills. This approach can enhance students’ self-esteem and independent thinking, while also encouraging them to provide active feedback on the learning process. Therefore, in practice, more targeted and effective teaching methods should be adopted to stimulate students’ enthusiasm and promote sustained participation in physical activities.

### 6.2 Research limitations

Despite this study’s foundation in Self-Determination Theory and Social Cognitive Theory to examine the influence of physical education teacher support on college students’ sports participation, several limitations should be acknowledged.

First, this study employed a cross-sectional design, which limits the ability to infer causal relationships between variables. As a result, the interpretation of predictive effects primarily relies on theoretical assumptions. Future research could employ longitudinal or experimental designs to further test the causal relationships among variables over time.

Second, the sample was drawn solely from college students in Shandong Province, which may affect the external representativeness and generalizability of the findings. Future studies are encouraged to broaden the sampling scope by including students from different regions and educational levels to improve the study’s applicability. In addition, given the potential impact of cultural background, cross-regional and cross-cultural comparative research would provide valuable insights.

Lastly, this study primarily focused on the role of teacher support and did not comprehensively examine other contextual factors, such as peer relationships, family environment, and campus sports resources, which may also affect students’ sports participation and psychological factors. Future research could expand the range of variables to reveal a more comprehensive and multilayered mechanism influencing college students’ sports participation, thereby offering more systematic theoretical support for optimizing physical education.

## 7 Conclusion

This study found that physical education teacher support, physical education learning motivation, self-efficacy, and sports participation were all significantly and positively correlated. Moreover, both physical education learning motivation and self-efficacy played key mediating roles in the relationship between teacher support and college students’ sports participation, including their independent mediating effects and their combined chain mediation effect. Based on these findings, physical education teachers should focus on creating a positive classroom atmosphere and providing timely, specific feedback to enhance students’ motivation and confidence. This, in turn, can encourage students to engage more actively in exercise, improve their ability perception, and experience the meaning and enjoyment of sports. Future research could further explore the roles of different influencing factors and cultural differences in sports participation and develop effective intervention measures. Additionally, course design should emphasize activity diversity by integrating knowledge, skills, games, and emotional components to strengthen students’ sense of value and positive emotional experience in sports, thereby better stimulating their enthusiasm for participation and motivation for long-term commitment.

## Data Availability

The raw data supporting the conclusions of this article will be made available by the authors, without undue reservation.
